# Synthesis, Characterization, and Properties of Novel Coplanar Bicyclic Compounds Based on Triazolofurazane Compounds

**DOI:** 10.3390/molecules30132803

**Published:** 2025-06-29

**Authors:** Mei-Qi Xu, Wen-Shuai Dong, Qamar-un-Nisa Tariq, Chao Zhang, Cong Li, Zu-Jia Lu, Bin-Shan Zhao, Qi-Yao Yu, Jian-Guo Zhang

**Affiliations:** State Key Laboratory of Explosion Science and Technology, Beijing Institute of Technology, Beijing 100081, China; xumeiqi1674@163.com (M.-Q.X.); dws08292020@163.com (W.-S.D.); qamarnisha@yahoo.com (Q.-u.-N.T.); 18810109953@126.com (C.Z.); l18656815056@163.com (C.L.); 15201657211@163.com (Z.-J.L.); 18810591681@163.com (B.-S.Z.)

**Keywords:** energetic materials, triazole, energetic salts, crystal structures, thermal analysis

## Abstract

In this study, a C-C bond-linked triazole-fused oxadiazole energetic compound, 4-amino-5-(4-amino-1,2,5-oxadiazol-3-yl)-2,4-dihydro-3*H*-1,2,4-triazol-3-one (**1**), was successfully designed and efficiently synthesized. Following nitration, a functional group-modified nitramine energetic compound (**2**) was obtained, and its energetic ionic salt (**3**) was further prepared. A comprehensive characterization of the structures of these three compounds was conducted, resulting in the successful elucidation of the single-crystal structures of compound **2**·Ca^2+^·6H_2_O and compound **3**·MeOH. Compound **2** exhibited a positive formation enthalpy (56.2 kJ·mol^−1^) and moderate mechanical sensitivity (FS = 120 N, IS = 12 J). Due to the presence of the nitramine group, compound 2 exhibited a relatively low thermal decomposition temperature (*T*_dec_ = 94 °C). However, the thermal stability of compound **3** was significantly improved (*T*_dec_ = 233 °C), which is attributed to salt formation. Compound **3** exhibits a positive formation enthalpy (121.0 kJ·mol^−1^), along with excellent detonation performance (D = 8120 m·s^−1^, P = 32.1 GPa) and reduced mechanical sensitivity (FS = 224 N, IS = 24 J). Therefore, the multi-heterocyclic compound, joined via C-C bond linkage, demonstrates outstanding performance, offering a new avenue for the design and synthesis of energetic materials.

## 1. Introduction

As a fundamental component of weaponry, energetic materials play a pivotal role in delivering effective damage in conventional weapon systems, serving as the primary source of initial energy. Numerous countries have placed significant emphasis on the research and application of novel energetic materials [1,2,3]. In recent years, the rapid advancement of these materials has been facilitated by the continuous integration of innovative design concepts and strategies, which have substantially enhanced the energy density of various energetic compounds [4,5]. Nevertheless, as the operational environments for munitions become increasingly demanding, the requirements for energy levels have escalated markedly, alongside a growing need for enhanced stability and safety [6,7,8]. Consequently, a key challenge in designing next-generation energetic molecules is enhancing their energy content while simultaneously applying rational molecular design principles and advanced synthetic techniques to satisfy the safety standards required for munition loading in weapon systems [9,10,11]. Traditional energetic compounds primarily release energy through the fracture of their scaffold structures and the decomposition of explosophoric groups, such as -NO_2_, -NH_2_, -N_3_, -ONO_2_, and -NNO_2_ [12,13]. However, an inherent contradiction exists between energetic properties and sensitivity; specifically, higher energetic performance is often directly associated with increased sensitivity [14,15].

The triazole ring possesses a high nitrogen content (60.8%), exhibits excellent stability, and can be readily chemically modified [16]. Heterocyclic structural motifs such as triazole and furazan exhibit enhanced physicochemical properties when interconnected through C-C bonds [17,18]. The introduction of energetic functional groups into these novel backbones significantly improves their density, enthalpy of formation, oxygen balance, and detonation performance [19,20]. The nitrogen and oxygen atoms present in these heterocycles possess high electronegativity, facilitating the formation of extensive π-conjugated systems similar to those found in benzene. This structural feature contributes to a reduction in the sensitivity of a compound while simultaneously improving its thermal stability. Furthermore, the high nitrogen content, coupled with relatively low carbon and hydrogen content, helps to achieve an optimal oxygen balance, which results in increased molecular density. Notably, the majority of decomposition products are nitrogen-based, thereby minimizing environmental pollution [21]. Furazan energetic compounds represent a distinctive class of chemicals that have garnered significant attention in the field of energetic materials due to their exceptional properties [22,23,24]. These compounds are primarily characterized by their high energy density, improved thermal stability, and insensitivity to mechanical stimuli. First, the high energy density of furazan ring compounds allows the rapid release of substantial amounts of energy during chemical reactions. In comparison to traditional energetic materials, furazan ring compounds can produce more energy for the same volume or mass, making them particularly advantageous in a wide range of applications. Additionally, furoxan ring compounds exhibit reduced sensitivity to shock, friction, and heat, thereby boosting their reliability and safety in practical applications [25]. Another remarkable feature of furazan ring compounds is their ability to undergo functionalization. Researchers can modify the physical and chemical properties of these compounds by introducing various functional groups, paving the way for the design and development of novel energetic materials [26,27]. This versatility allows for the tailoring of compounds to meet specific performance requirements. In summary, furazan ring energetic compounds have become a crucial focus in modern energetic materials research due to their high energy density, excellent thermal stability and mechanical properties, diverse functionalization capabilities, and favourable environmental profiles [28]. With ongoing research, the development of new furazan ring compounds is anticipated to expand, opening new avenues for advancements and applications in energetic materials. Energetic polycyclic azole compounds that are high in nitrogen consist of two or more nitrogen-containing heterocycles. Research has shown that bicyclic and polycyclic azoles can be synthesized from monocyclic azoles, offering more nitrogen atoms and modification sites for improved performance [29]. Numerous high-performance energetic bicyclic and polycyclic azole compounds have been synthesized worldwide, displaying diverse properties influenced by the connectivity of their rings [30]. The combination of triazole and furazan via C-C bonding unites their energy advantages, thus improving the overall performance of the target compounds. This molecular design approach has enabled the synthesis of novel polycyclic high-nitrogen azole compounds, representing a significant advancement in research on energetic materials with promising practical applications.

In this work, we designed and synthesized a new class of nitrogen-rich compounds known as triazolofurazan by combining different structural units within a single molecule to leverage the complementary advantages of heterocycles. The furazan ring with an amino oxime structure was synthesized through a cyclization reaction starting from malononitrile. Subsequently, the precursor of the 1,2,4-triazole-furazan skeleton was formed through a cyclization reaction between the amino oxime group and urea [31]. The resulting structures were thoroughly characterized using single-crystal X-ray diffraction, infrared spectroscopy (IR), elemental analysis (EA), differential scanning calorimetry (DSC), thermogravimetric analysis (TG), and various performance tests. Additionally, relevant theoretical calculations were conducted to explore the relationship between structural properties and molecular stability. The detonation performance and sensitivity of these energetic compounds were also examined, providing valuable insights into the advancement of next-generation multi-nitrogen heterocyclic energetic compounds.

## 2. Results and Discussion

### 2.1. Synthesis of Energetic Compounds

**Scheme 1:**
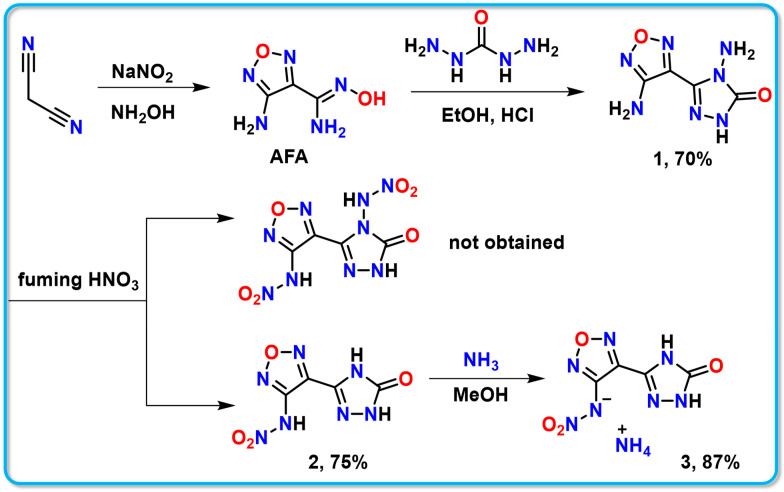
Synthesis of energetic compounds **1**–**3.**

The triazolofurazane compound **1** (4-amino-5-(4-amino-1,2,5-oxadiazol-3-yl)-2,4-dihydro-3*H*-1,2,4-triazol-3-one) was synthesized through a simple and straightforward three-step reaction using malononitrile as a starting material [32]. The intention was to synthesize dinitroamino compounds via a nitration reaction. However, during this process, the nitro group on the triazole underwent hydrolysis, leading to the formation of compound **2** (N-(4-(5-oxo-4,5-dihydro-1*H*-1,2,4-triazol-3-yl)-1,2,5-oxadiazol-3-yl) nitramide) (Scheme 1).

### 2.2. X-Ray Structure Analysis

The crystal structures of **2**·Ca^2+^·6H_2_O and **3**·MeOH were confirmed by X-ray single-crystal diffraction at 116 K. The detailed crystallographic data for both crystal structures are listed in Table 1, and the complete information on bond lengths and bond angles is provided in the Supporting Information.

**Figure 1:**
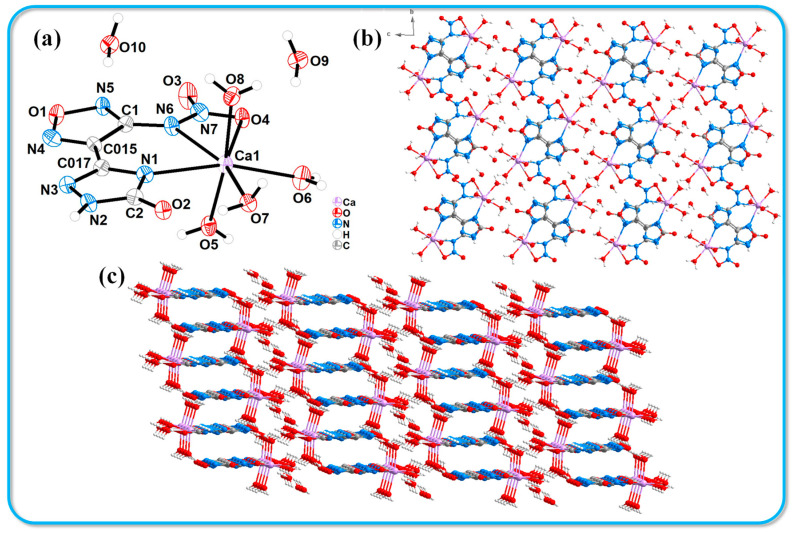
Crystal structure of **2**·Ca^2+^·6H_2_O. (**a**) Thermal ellipsoids are set at a 50% probability level. (**b**,**c**) Crystal stacking diagrams of **2**·Ca^2+^·6H_2_O.

An adequate quantity of compound **2** was dissolved in 5 mL of a 1:1 methanol/calcium carbonate solution and subsequently kept under controlled temperature and humidity conditions for two weeks, resulting in the formation of yellow block-shaped crystals. High-quality crystals with optimal dimensions were carefully selected for X-ray single-crystal diffraction analysis. Despite numerous attempts to cultivate single crystals using deionized water, we were unsuccessful in obtaining crystals suitable for testing. The crystallographic information of the resulting compound **2**·Ca^2+^·6H_2_O is presented in Table 1. This compound belongs to the triclinic crystal system with a *P*-1 space group and contains two molecules per unit cell. The crystal density (1.732 g·cm^−3^) was measured at 116 K. Figure 1 illustrates the crystal structure and stacking arrangement of compound **2**·Ca^2+^·6H_2_O. In Figure 1a, it is observed that the calcium ion (Ca1) is six-coordinated, engaging in coordination with nitrogen and oxygen atoms from the host molecules. The bond lengths for Ca1-N1, Ca1-N6, and Ca1-O4 are 2.545(4) Å, 2.477(4) Å, and 2.643(4) Å, respectively. Additionally, Ca1 forms coordination bonds with the oxygen atoms of three water molecules. Figure 1b,c present the crystal stacking diagrams of the molecular structure as viewed along the a-axis and b-axis, respectively. It is clear from these diagrams that compound **2**·Ca^2+^·6H_2_O exhibits a zero-dimensional structure. The molecular stacking observed from the c-axis reveals a face-to-face layered arrangement, with a layer spacing of 3.32 Å.

**Figure 2:**
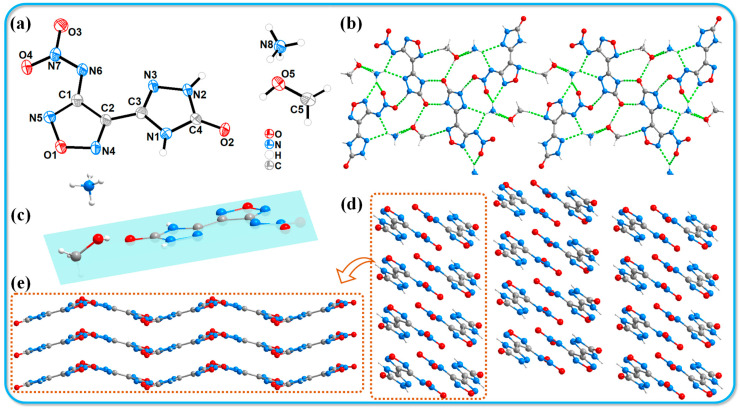
Crystal structure of **3**·MeOH. (**a**) Thermal ellipsoids are set at a 50% probability level. (**b**) Hydrogen bond network **3**·MeOH. (**c**) Structural planarity of **3**·MeOH. (**d**,**e**) Crystal stacking diagrams of **3**·MeOH.

**Figure 3:**
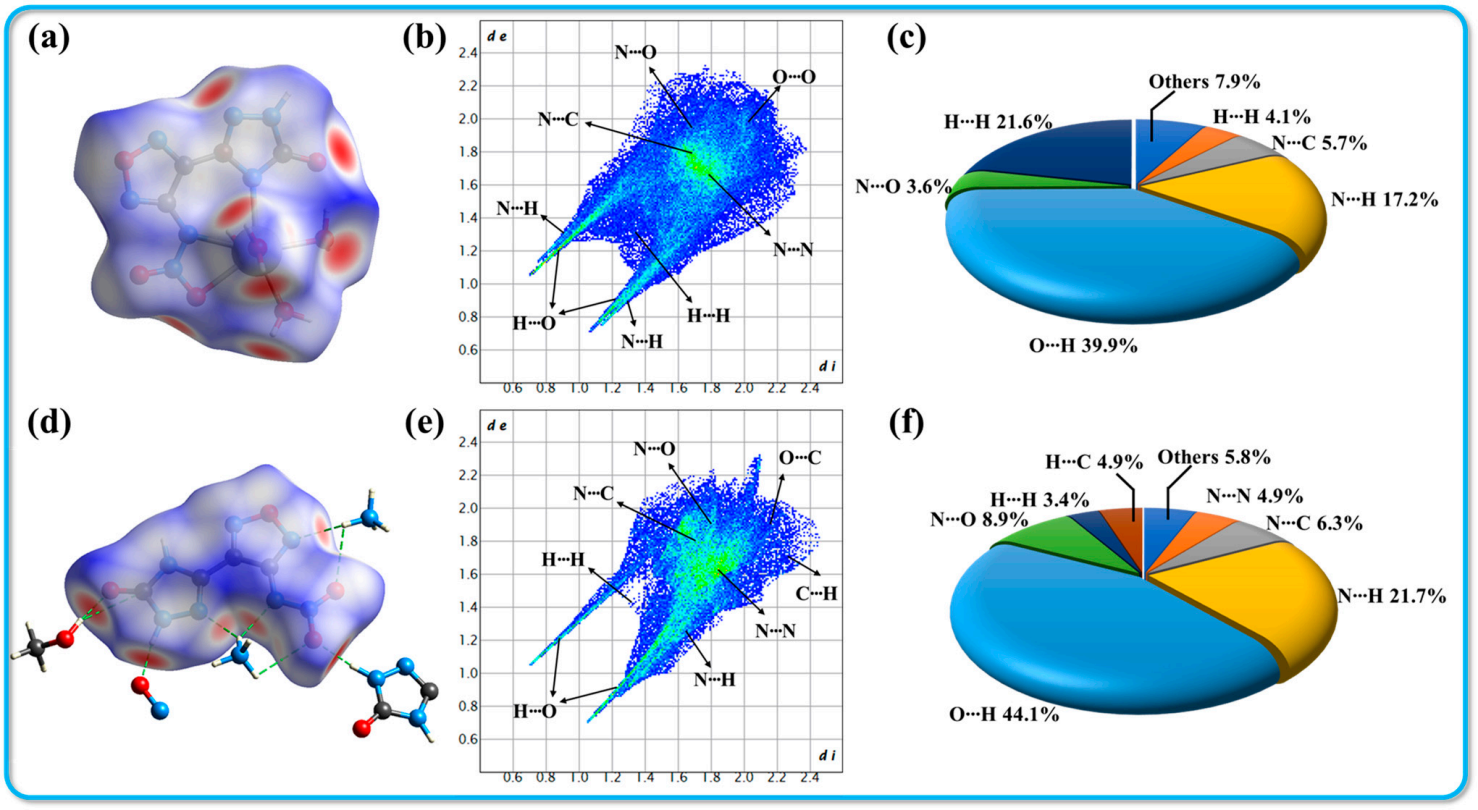
Hirshfeld surfaces (**a**,**d**), 2D fingerprint plots (**b**,**e**), and atomic-to-atomic contact percentage contribution (**c**,**f**) for crystals **2**·Ca^2+^·6H_2_O and **3**·MeOH, respectively.

An appropriate amount of compound **3** was dissolved in 5 mL of a 1:1 methanol/water solution and allowed to slowly evaporate over a period of two weeks under controlled temperature and humidity conditions, resulting in the formation of yellow block-shaped crystals. An appropriately sized crystal was chosen for X-ray single-crystal diffraction analysis. The labeled single-crystal structure and the crystal stacking diagram are shown in Figure 2. The specific crystallographic data are summarized in Table 1. Compound **3** crystallizes in the monoclinic system with a space group of *P*2_1_/n and includes four molecules per unit cell. The crystal density (1.630 g·cm^−3^) was determined at 116 K. Figure 2a shows the thermal ellipsoid diagram of the single molecule, indicating that the methanol molecules co-crystallize with the host molecules during the crystallization process. The presence of ammonium cations and methanol molecules significantly expands the hydrogen bond network within the molecular structure, enhancing the diversity of hydrogen bonds. The following hydrogen bonds have been identified in the molecular framework: N1-H1···O2 (2.81 Å), N2-H2···O3 (2.77 Å), O5-H5···O2 (2.83 Å), N8-H8A···O5 (2.96 Å), N8-H8B···O5 (2.84 Å), N8-H8C···C···N3 (3.07 Å), N8-H8C···N6 (2.92 Å), N8-H8D···O4 (3.00 Å), N8-H8D···N5 (3.10 Å), and C5-H5C···N4 (3.45 Å). The diverse types of hydrogen bonds connect the molecules to form a one-dimensional chain structure, as illustrated in Figure 2b. The host molecule exhibits notable planarity (see Figure 2c). Figure 2d,e depict the stacking structure of compound **3**, which demonstrates a wave-like stacking arrangement. This particular stacking configuration effectively reduces the mechanical sensitivity of the compound, consistent with the experimental observations. In addition, we used CrystalExplorer software (v21.5) [33] to draw Hirshfeld surface and 2D fingerprint plots (shown in Figure 3) and analyzed the interaction forces between molecules, including hydrogen bonds and π-π interactions. The details are shown in the Supporting Information (Section S3).

**Figure 4:**
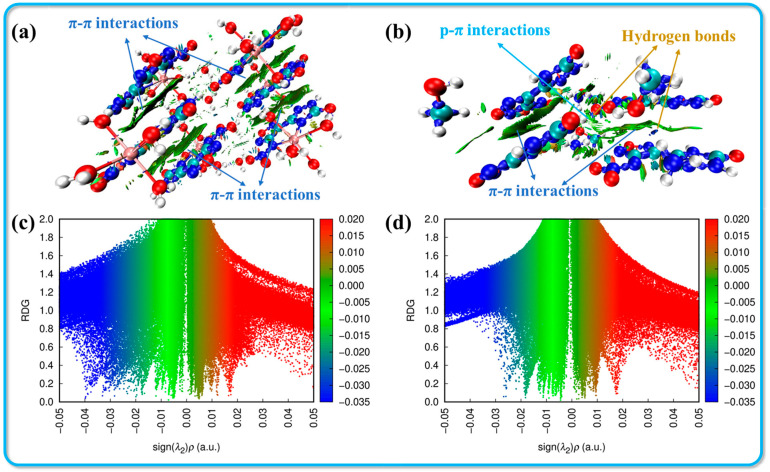
Non-covalent interaction analysis for crystals **2**·Ca^2+^·6H_2_O (**a**,**c**) and **3**·MeOH (**b**,**d**) (red: strong repulsion; blue: strong attraction; and green: weak interaction).

To provide a clearer depiction of the non-covalent interactions present in these crystal structures, the analysis includes calculating non-covalent interactions (NCIs) using the gradient isosurface method. As suggested by Johnson et al., the reduced density gradient (RDG) isosurface, colored according to the NCI diagram, enables the identification of different types of forces present through distinct color regions. The results obtained from Multiwfn and VMD are presented in Figure 4 [34]. When combined with the Hirshfeld surface analysis, it becomes straightforward to identify the blue and green small ovals in the NCI image, which correspond to hydrogen bonds. These regions signify a high electron density, where dark blue areas indicate stronger attractive interactions. This observation confirms the presence of significant hydrogen bonding between the hydrogen atom in the cation and the oxygen or nitrogen atoms in the anion. Furthermore, owing to the planar configuration and layered stacking structure observed in both crystal systems, the large green areas between adjacent parallel molecules suggest a rich presence of π-π interactions within the structure. These interactions contribute to enhanced molecular stability and reduced sensitivity of the compounds, emphasizing the importance of non-covalent interactions in determining the physical properties of the crystals.

The structures of compounds **2** and **3** were optimized using Gaussian 16 software at the B3LYP/6-311+G(d,p) level of theory. Following optimization, various properties of the structures were calculated and analyzed using a combination of Multiwfn and VMD, focusing on local molecular planarity, electrostatic potential (ESP), local orbital analysis (LOL-π), and the isochemical shielding surface (ICSS). To quantitatively determine the planarity of the molecules, two key parameters were utilized: the molecular planarity parameter (MPP) and the deviation plane span (SDP) [35]. These parameters serve to characterize the extent to which the molecular structure adheres to a planar configuration.

**Figure 5:**
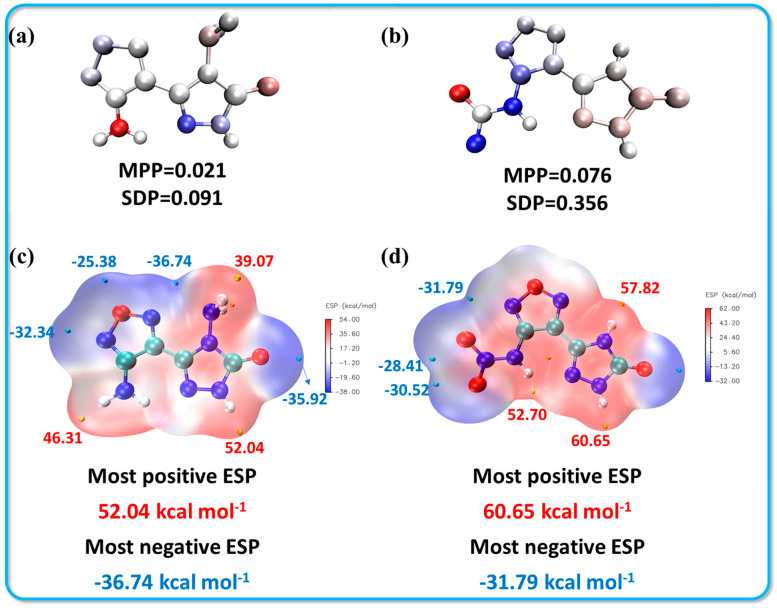
Molecular planarity and electrostatic potential plots for compounds **1** (**a**,**c**) and **2** (**b**,**d**).

Molecular planarity parameter (MPP): This parameter reflects the overall planarity of the region under consideration. Smaller MPP values indicate greater planarity, with values approaching zero indicating a fully planar structure. Deviation plane span (SDP): This measures the maximum deviation from the fitted plane within the analyzed area. Essentially, the SDP quantifies how far the structure extends perpendicularly from this fitting plane. Like the MPP, a smaller SDP value suggests that the structure is closer to being planar. In summary, both the MPP and SDP provide valuable quantitative metrics for evaluating the planarity of the optimized structures of compounds **1** and **2**. An ideally planar structure would have both parameters equal to zero, indicating minimal deviation and a strong planar configuration. Analyzing these aspects helps in understanding the stability and potential reactivity of the molecules based on their conformational characteristics. As illustrated in Figure 5a,b, the molecular planarity parameter (MPP) for compound **1** is notably closer to zero (MPP = 0.021, SDP = 0.091), indicating a more striking planarity compared to compound **2**. While compound **2** exhibits a similar MPP value (MPP = 0.097), it has a significantly larger deviation plane span (SDP = 0.356). This increased SDP is primarily attributed to the deflection of the N-NO_2_ group, which is consistent with the observed crystal structure data. In addition to planarity, the electrostatic potential (ESP) surfaces for both compounds were calculated and are shown in Figure 5c,d [36]. The colors on the ESP surface represent different values of potential energy, with orange and cyan spheres indicating the maximum and minimum ESP values, respectively. For both compounds, the highest potential energy values were located at the N-H position of the triazole moiety, with compound **1** having a value of 52.04 kcal·mol^−1^ and compound **2** showing a higher value of 60.65 kcal·mol^−1^. The presence of a larger positive ESP value and a more extensive electropositive region (depicted in red) typically correlates with an increased sensitivity in energetic compounds. This observation suggests that compound **2**, having a higher ESP and broader electropositive area, may exhibit greater sensitivity, aligning with the experimental results observed in sensitivity tests. This correlation underscores the importance of molecular geometry and electrostatic properties in determining the reactivity and stability of energetic materials.

Aromatic molecules exhibit distinct characteristics across various dimensions, including energy, structure, reactivity, magnetic properties, and electronic structure. A comprehensive statistical analysis of a multitude of aromatic systems reveals that these compounds consistently display several interrelated features, including bond length equalization, significant overall electron delocalization, the formation of an induced ring current in the presence of an external magnetic field, substantial delocalization energy, and enhanced structural stability. Consequently, the details of any single aspect of a molecule can provide insights into its aromaticity. However, it is important to note that the relationships among these diverse characteristics do not always conform to established norms, a phenomenon referred to as the multidimensionality of aromaticity. Therefore, to achieve a nuanced and accurate description of aromaticity, it is essential to consider multiple aromaticity indices that reflect various molecular properties concurrently. Among these measures, the isochemical shielding surface (ICSS) has emerged as the most widely utilized index for assessing aromaticity.

**Figure 6:**
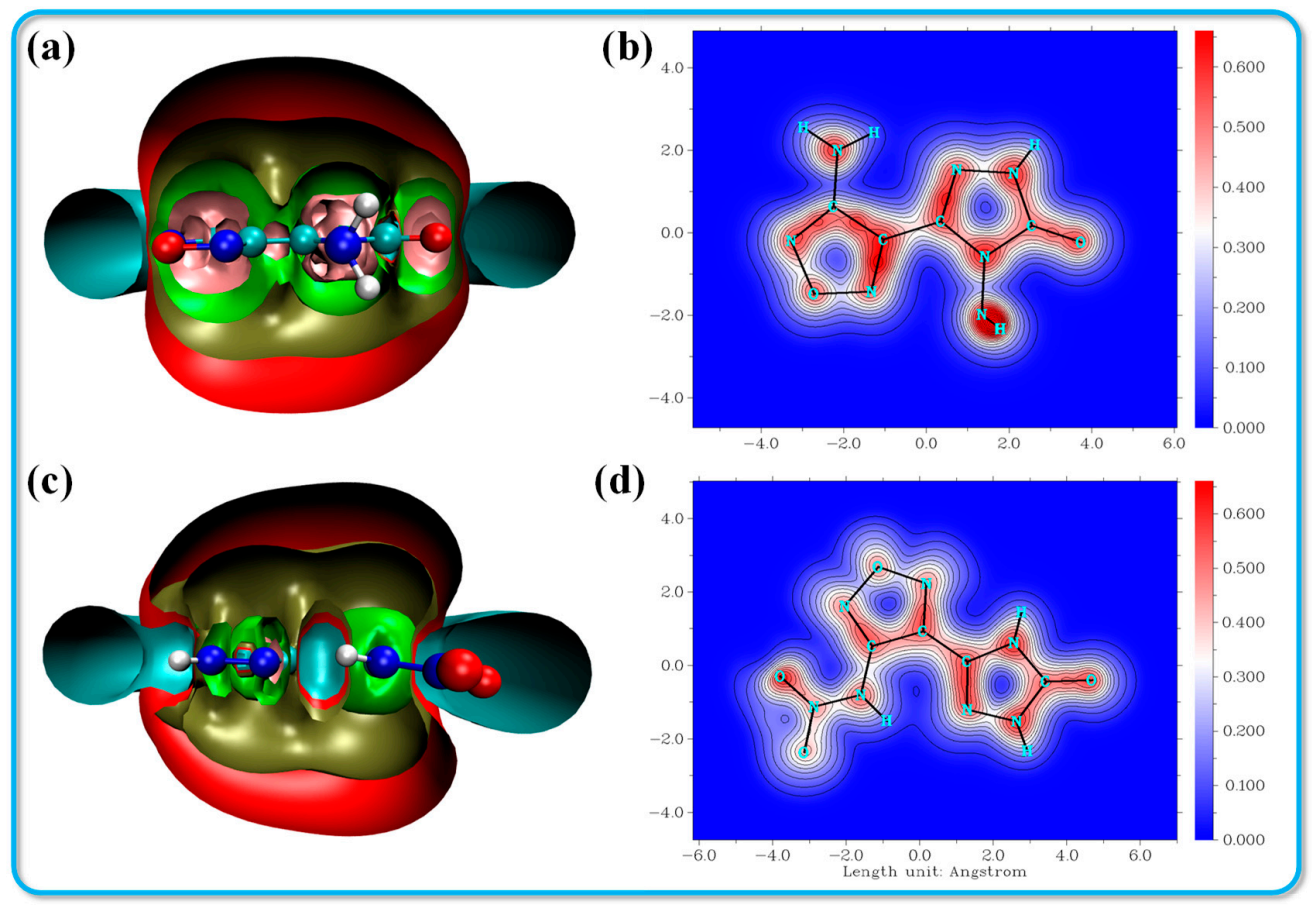
ICSS isosurface analysis and LOL-π color-filled graph for crystals **2** (**a**,**b**) and **3** (**c**,**d**).

This indicates that the negative values assigned to the magnetic shielding parameters, which are not centered at the nucleus, are artificially determined. A more negative shielding value reflects stronger magnetic field shielding, which is indicative of enhanced aromaticity [37,38]. As illustrated in Figure 6a, the ICSS cutting plane reveals a pink region on the shielding surface of compound **1**, suggesting that it exhibits strong aromaticity. Both compounds display near-planar molecular structures; however, the ICSS cutting plane for compound **2** shows a slight displacement of the N-NO_2_ group relative to the parent ring. Furthermore, the Localized Orbital Locator for π (LOL-π) is another significant index used to assess the stability of the π electron distribution within the compounds, as depicted in Figure 6b,d. The isosurface value for LOL-π is set at 0.4. The π electrons in both compounds **1** and **2** demonstrate a uniform distribution across the triazole ring, furazan ring, and azide group. Notably, the red region in compound **1** is darker, indicating a robust conjugated system. In contrast, the initial color of compound **2** at the N-NO_2_ position is lighter, signifying weaker conjugation in that area.

### 2.3. Energetic Performance and Safety

**Figure 7:**
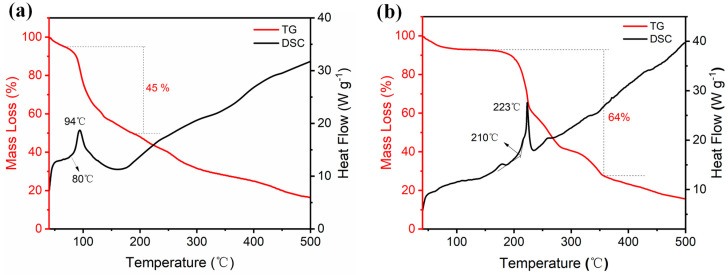
DSC and TG curves for **2** (**a**) and **3** (**b**).

Thermal stability is a fundamental evaluation index for assessing the safety performance of energetic materials. In this study, the thermal stability of compound **2** and ammonium salt **3** was investigated using differential scanning calorimetry (DSC) and thermogravimetric analysis (TGA) at a heating rate of 10 °C·min^−1^ in a nitrogen atmosphere. A sample weight of 0.5 mg was utilized for each test. The results from the DSC and TGA analyses are presented in Figure 7. According to the DSC thermogram, compound **2** undergoes exothermic decomposition starting at 80 °C, with the maximum exothermic peak appearing at 94 °C. The corresponding TG curve indicates a mass loss of 45%. Based on its thermal decomposition behavior, it can be concluded that the analyzed powder sample did not contain any water molecules from the solvent. After nitration with fuming nitric acid, the decomposition temperature decreased significantly; however, the thermal stability improved markedly upon conversion to the ammonium salt. For ammonium salt **3**, the initial exothermic decomposition peak was found at 210 °C, and the maximum exothermic peak temperature reached 223 °C. This decomposition process involved a substantial mass loss of 65%. These results emphasize the important role of chemical modifications in improving the thermal stability of energetic materials.

The standard enthalpy of formation of energetic compounds is a crucial parameter for evaluating their energy content. For high-nitrogen energetic materials, the energy is derived from their explosive functional groups, and their detonation performance is influenced by a high positive enthalpy of formation. According to its definition, the standard enthalpy of formation refers to the enthalpy change that occurs when explosive compounds are synthesized from their stable elemental forms under standard conditions. This measurement is fundamental to the theoretical calculation of various detonation performance parameters of explosives. In this study, the standard enthalpies of formation for compounds **2** and **3** were determined using Gaussian 16 software. Geometric optimization and vibrational analysis were performed at the B3PW91/6-31G** level, followed by a higher-precision single-point energy calculation at the M062X/def2TZVP level. The enthalpies of formation were calculated using the atomization energy method. The results showed that both compounds exhibited positive enthalpies of formation, with values of 56.2 kJ·mol^−1^ for compound **2** and 121.0 kJ·mol^−1^ for compound **3**. These positive values suggest that both compounds possess considerable energy content, which is essential for their potential use in applications requiring high energy output, such as explosives. The comparison of these values also provides insights into the relative stability and energetic potential of the compounds, aiding in the evaluation of their performance in practical applications.

The density of energetic materials plays a critical role in determining their detonation performance. In this study, the densities of compounds **2** and **3** were measured as approximately 1.60 g·cm^−3^ and 1.64 g·cm^−3^, respectively, which are comparable to that of TNT (1.63 g·cm^−3^). Using these density values and the calculated standard enthalpies of formation, the detonation performance of the compounds was evaluated with EXPLO 5 (v6.04), and the results are summarized in Table 2. The results indicate that both compounds exhibit relatively high detonation performance, with detonation velocities ranging from 7689 m·s^−1^ to 8120 m·s^−1^ and detonation pressures between 28.9 GPa and 32.1 GPa. Notably, compared to the traditional explosive TNT, which has a detonation velocity of 6881 m·s^−1^ and a detonation pressure of 19.5 GPa, compound **3** shows a significant improvement, achieving a detonation velocity of 8120 m·s^−1^ and a detonation pressure of 32.1 GPa. Furthermore, the mechanical sensitivities of the compounds were measured using the BAM standard method, and the results are presented in Table 2. The friction sensitivity (FS) and impact sensitivity (IS) of compound **2** were found to be 120 N and 12 J, respectively. In contrast, compound **3** exhibited a higher friction sensitivity of 224 N and impact sensitivity of 24 J. The reduced mechanical sensitivity of compound 3 can be attributed to the close packing of its molecular structure and the presence of extensive hydrogen bonding interactions, which enhance its stability. Overall, the findings suggest that compounds **2** and **3** not only demonstrate promising detonation performance compared to traditional explosives like TNT but also exhibit different mechanical sensitivities that could influence their safety and application in practical scenarios.

## 3. Materials and Methods

***Caution*!** All the nitrogen-rich compounds used in this study are energetic materials and may explode under certain conditions. Appropriate safety precautions must be taken during preparation and/or handling. Only small quantities should be prepared and studied. The reagents used in this work are all analytically pure and do not require further purification.

### 3.1. Reagents and Instruments

**Reagents**: malononitrile, sodium nitrite, 50% hydroxylamine, ammonia solution, and carbohydrazide were purchased from Shanghai Aladdin Biochemical Technology Co., Ltd., Shanghai, China. Ethanol and methanol were purchased from Shanghai Titan Scientific Co., Ltd., Shanghai, China. Hydrochloric acid was purchased from Beijing Tong Guang Fine Chemicals Company, Beijing, China.

**Instruments**: ^1^H and ^13^C spectra were recorded using a 400 MHz nuclear magnetic resonance spectrometer (Bruker AVANCE NEO 400) at frequencies of 400.13 MHz and 100.62 MHz, respectively (Bruker, Bremen, Germany). Elemental analyses were performed on a Flash EA 1112 fully automatic trace element analyzer (Waltham, MA, USA). The FT-IR spectra were recorded as KBr pellets on a Bruker Equinox 55 (Bruker, Bremen, Germany). Mass spectra were recorded on a Thermo-QE plus (Thermo Fisher, Waltham, MA, USA). Single-crystal X-ray diffraction analysis was carried out on a Bruker CCD area detector diffractometer (Bruker, Bremen, Germany). Thermal analysis tests were performed on a DSC-800B differential scanning calorimeter (Innuo, Shanghai, China). The impact and friction sensitivities were measured using a standard BAM fall hammer and a BAM friction tester, respectively (OZM, Prague, Czech Republic).

### 3.2. Synthesis of 4-Amino-5-(4-amino-1,2,5-oxadiazol-3-yl)-2,4-dihydro-3H-1,2,4-triazol-3-one (**1**)

In a 500 mL single-necked flask, malononitrile (10.00 g, 151 mmol) and water (200 mL) were added and stirred for 5 min. Sodium nitrite (11.80 g, 171 mmol) was slowly added at 0 °C, and then, 8.3 mL hydrochloric acid (12 N) was added dropwise to the above mixture solution. After maintaining the temperature for 15 min, the ice bath was removed, and the reaction was continuously stirred at room temperature for 1.5 h. The reaction mixture was cooled to 0 °C, and then, a 50% hydroxylamine aqueous solution (30.90 g, 468 mmol) was added. After removing the ice bath, the reaction was carried out at room temperature for 1 h, and the reaction mixture was refluxed at 100 °C for 2 h. Upon cooling to room temperature, the reaction mixture was placed in an ice bath at 0 °C, and hydrochloric acid was added slowly dropwise to adjust the pH to 7.0. As hydrochloric acid was added, a solid precipitated from the mixture. Finally, the precipitate was filtered, washed with water, and dried, yielding 17.50 g of an intermediate product. (*Z*)-4-amino-N′-hydroxy-1,2,5-oxadiazole-3-carboximidamide was obtained in 80% yield. In a 50 mL single-necked flask, (*Z*)-4-amino-N′-hydroxy-1,2,5-oxadiazole-3-carboximidamide (1.00 g, 7 mmol) and carbohydrazide (DAU) (0.63 g, 7 mmol) were added in batches to a solution of ethanol/water (1:1, 15 mL). Hydrochloric acid (12 N, 1 mL) was added slowly, resulting in a pale yellow solution. The reaction mixture was then heated to 85 °C for 12 h before being cooled to room temperature. At the bottom of the reaction mixture, a white solid had settled, and the top layer was an orange liquid. The precipitate was filtered and dried to obtain 4-amino-5-(4-amino-1,2,5-oxadiazol-3-yl)-2,4-dihydro-3*H*-1,2,4-triazol-3-one (**1**), with the following yield: 0.90 g, 70%. IR (cm^−1^) ν = 3448, 3392 (-NH_2_), 3361(-NH_2_), 3284 (-NH), 1726, 1702, 1637, 1596, 1548, 1518, 1381, 1343, 1186, 1111, 1003, 976, 890, 856, 840, 810, 789, 748, 735, 700, 686, 673, 662, 649, 630, and 615. Elemental analysis calcd (%) for C_4_H_5_N_7_O_2_ (183.05): C 26.23, H 2.75, N 53.54. found: C 26.24, H 2.77, N 53.56.

### 3.3. Synthesis of N-(4-(5-oxo-4,5-dihydro-1H-1,2,4-triazol-3-yl)-1,2,5-oxadiazol-3-yl)nitramide (**2**)

In an ice bath at 0 °C, 4-amino-5-(4-amino-1,2,5-oxadiazol-3-yl)-2,4-dihydro-3*H*-1,2,4-triazole-3-one (0.50 g, 2.7 mmol) was slowly added to 3 mL of 98% HNO_3_. After a reaction period of 0.5 h, the reaction mixture was allowed to warm to room temperature and stirred for an additional 2 h. Upon completion of the reaction, the mixture was poured into ice water and extracted with ethyl acetate (3 × 30 mL). The organic phase was dried over anhydrous sodium sulfate and concentrated under reduced pressure to give a light-yellow solid **2**, with a yield of 0.43 g (75%). IR (cm^−1^) ν = 3332 (-NH), 2795, 1690, 1648, 1603 (-NO_2_), 1560, 1517, 1458, 1438, 1326, 1304 (-NO_2_), 1153, 1096, 1028, 964, 917, 903, 862, 806, 770, 720, 707, 659, 645, and 612. Elemental analysis calcd (%) for C_4_H_3_N_7_O_4_ (213.02): C 22.54, H 1.42, N 16.01. found: C 22.52, H 1.44, N 16.03.

### 3.4. Synthesis of Ammonium Nitro(4-(5-oxo-4,5-dihydro-1H-1,2,4-triazol-3-yl)-1,2,5-oxadiazol-3-yl)amide (**3**)

Aqueous ammonia was added dropwise to a 5 mL methanol solution of compound **2** (0.21 g, 1 mmol), and the mixture was stirred at room temperature for 12 h. The resulting precipitate was filtered and dried to obtain a yellow solid **3**. Yield: 0.20 g, 87%. IR (cm^−1^) ν = 3211 (-NH), 1703, 1519 (-NO_2_), 1495, 1396, 1273 (-NO_2_), 1188, 1084, 1037, 1010, 961, 925, 889, 876, 831, 792, 781, 754, and 726.

## 4. Conclusions

Two novel nitrogen-rich energetic compounds, designated as compounds **1** and **2**, were designed and synthesized based on the structural framework of triazole-linked furazan compounds. During the nitration process of compound **1**, a hydrolysis reaction was observed, leading to the formation of compound **2**. Additionally, single crystals of **2**·Ca^2+^·6H_2_O and **3**·MeOH were successfully grown, and their crystal structures were characterized using single-crystal X-ray diffraction. Further computational simulations were conducted to systematically investigate the weak interactions and hydrogen bonding networks within these structures. The results revealed that the planar conjugated cation structure, extensive hydrogen bonding, and widespread π-π interactions collectively endowed these compounds with excellent physicochemical properties and detonation performance. The thermal stability of compounds **2** and **3** was thoroughly analyzed. The results indicated that ammonium salt **3** exhibited an initial exothermic decomposition peak at 210 °C, with a maximum exothermic decomposition temperature of 223 °C, demonstrating good thermal stability. In contrast, compound **2** showed a decomposition onset temperature of 80 °C, with an exothermic peak at 94 °C, indicating relatively poor thermal stability. This suggests that the formation of energetic salts significantly improves the thermal stability of energetic compounds. In terms of detonation performance, both compounds exhibited high performance, with detonation velocities ranging from 7689 to 8120 m·s^−1^ and detonation pressures ranging from 28.9 to 32.1 GPa. Notably, the detonation velocity (8120 m·s^−1^) and detonation pressure (32.1 GPa) of compound **3** were significantly higher than those of TNT (6881 m·s^−1^ and 19.5 GPa, respectively). Furthermore, the mechanical sensitivities of the compounds were evaluated using the BAM standard method. Compound **2** exhibited a friction sensitivity (FS) of 120 N and an impact sensitivity (IS) of 12 J, while compound **3** was markedly less sensitive to mechanical stimuli (FS = 224 N; IS = 24 J), indicating that salt formation substantially lowers the mechanical sensitivity of energetic compounds. In conclusion, the results of this study demonstrate that triazole-linked furazan energetic salts not only possess excellent detonation performance but also exhibit high safety, providing important theoretical and practical guidance for the design and development of novel high-energy-density energetic materials.

## Figures and Tables

**Table 1:** Crystal data and structure refinement for **2**·Ca^2+^·6H_2_O and **3**·MeOH.

Compounds	2·Ca^2+^·6H_2_O	3·MeOH
Empirical formula	C_4_H_13_CaN_7_O_10_	C_5_H_10_N_8_O_5_
Formula weight	359.29	262.21
Temperature/K	116	116
Crystal system	triclinic	monoclinic
Space group	*P*-1	*P*2_1_/n
a/Å	6.8232(14)	4.1568(6)
b/Å	9.6903(19)	12.7839(19)
c/Å	10.997(2)	20.122(3)
α/°	81.535(17)	90.00
β/°	76.670(18)	92.021(13)
γ/°	78.334(17)	90.00
Volume/Å^3^	689.0(2)	1068.6(3)
Z	2	4
ρ_calc_g/cm^3^	1.732	1.630
μ/mm^−1^	0.524	0.143
F(000)	372	544
Crystal size/mm^3^	0.23 × 0.15 × 0.15	0.35 × 0.13 × 0.09
2θ range for data collection/°	6.06 to 52	6.38 to 52
Index ranges	−7 ≤ h ≤ 8, −11 ≤ k ≤ 9, −13 ≤ l ≤ 11	−5 ≤ h ≤ 5, −15 ≤ k ≤ 11, −20 ≤ l ≤ 24
Reflections collected	4599	4251
Independent reflections	2618 [R_int_ = 0.0546 (inf-0.9 Å)]	2060 [R(int) = 0.0698 (inf-0.9 Å)]
Data/restraints/parameters	2618/22/235	2060/0/166
F^2^	1.045	1.048
Final R indexes [I ≥ 2σ (I)]	R_1_ = 0.0697, wR_2_ = 0.1495	R_1_ = 0.0729, wR_2_ = 0.1681
Final R indexes	R_1_ = 0.1090, wR_2_ = 0.1876	R_1_ = 0.1254, wR_2_ = 0.2059
CCDC	2441168	2441167

**Table 2:** Energetic and physicochemical properties of **2** and **3**.

Compounds	*ρ* ^a^ [g·cm^−3^]	*T*_d_ ^b^ [°C]	Δ*H*_f_^c^ [kJ·mol^−1^]	*D* ^d^ [m·s^−1^]	*P* ^e^ [GPa]	*IS* ^f^ [J]	*FS* ^g^ [N]
**2**	1.60	94	56.2	7689	28.9	12	120
**3**	1.64	233	121.0	8120	32.1	24	224
**TNT**	1.65	295	−67.0	6881	19.5	353	36.5

^a^ Measured density. ^b^ Thermal decomposition temperature. ^c^ Enthalpy for formation. ^d^ Detonation velocity. ^e^ Detonation pressure. ^f^ Impact sensitivity. ^g^ Friction sensitivity.

## Data Availability

CCDC 2441168 and 2441167 contain supplementary crystallographic data for this paper. These data can be obtained free of charge via https://www.ccdc.cam.ac.uk/structures/ (accessed on 26 June 2025) (or from the Cambridge Crystallographic Data Centre, 12, Union Road, Cambridge CB2 1EZ, UK; fax: +44 1223 336033).

## Supplementary Materials

The following supporting information can be downloaded at https://www.mdpi.com/article/10.3390/molecules30132803/s1, Section S1. Crystal structure data: Tables S1–S4: Bond lengths and bond angles; Section S2. Theoretical calculation method for formation enthalpy; Section S3: Intermolecular interactions; Section S4. NMR spectra: Figures S1–S6: ^1^H and ^13^C NMR spectra for all compounds; Section S5. IR spectra: and Figures S7–S9: IR spectra of compounds **1**–**3**. Section S6. Noncovalent interaction analysis. Figure S10. Noncovalent interaction analysis for **2**·Ca^2+^·6H_2_O (left) and **3**·MeOH (right).

## References

[1] Fershtat L.L., Makhova N.N. (2020). 1,2,5-Oxadiazole-based high-energy-density materials: Synthesis and performance. ChemPlusChem.

[2] Vinogradov D.B., Fershtat L.L. (2024). Energetic azine N-oxides: State-of-the-art achievements in the synthesis and performance. Chem. Eng. J..

[3] Dong W.-S., Zhang P.-P., Xu M.-Q., Lu Z.-J., Li Z.-M., Wang K., Yu Q.-Y., Zhang J.-G. (2025). One-pot synthesis of innovative multicomponent complexes as catalysts for enhanced decomposition of ammonium perchlorate. Small.

[4] Fischer D., Klapotke T.M., Stierstorfer J. (2015). 1,5-Di(nitramino)tetrazole: High sensitivity and superior explosive performance. Angew. Chem. Int. Ed..

[5] Yadav A.K., Ghule V.D., Dharavath S. (2023). Thermally stable and insensitive energetic cocrystals comprising nitrobarbituric acid coformers. Cryst. Growth Des..

[6] Tang Y., He C., Imler G.H., Parrish D.A., Shreeve J.M. (2018). Ring closure of polynitroazoles via an *N*,*N*′-alkylene bridge: Towards high thermally stable energetic compounds. J. Mater. Chem. A.

[7] Chang J., Xiong J., Jia H., He C., Pang S., Shreeve J.M. (2023). Polyiodo azole-based metal–organic framework energetic biocidal material for synergetic sterilization applications. ACS Appl. Mater. Interfaces.

[8] Fischer D., Klapotke T.M., Stierstorfer J. (2014). Potassium 1,1′-dinitramino-5,5′-bistetrazolate: A primary explosive with fast detonation and high initiation power. Angew. Chem. Int. Ed..

[9] Hu L., Staples R.J., Shreeve J.M. (2021). Energetic compounds based on a new fused triazolo[4,5-d]pyridazine ring: Nitroimino lights up energetic performance. Chem. Eng. J..

[10] Zhang J., Zhang J., Singh J., Wu W., Staples R.J., Zhang J., Shreeve J.M. (2024). Highly stable poly-nitro components achieved through supramolecular encapsulation. J. Mater. Chem. A.

[11] Qin Y., Yang F., Chen Z., Lu M., Wang P. (2024). Revealing the electro-oxidation mechanism of 5-aminotetrazole on nickel-based oxides and synthesizing 5,5′-azotetrazolate salts. Inorg. Chem..

[12] Xu M., Dong W.-S., Tariq Q.-u.-N., Lu Z.-J., Li D.-K., Yu Q., Zhang J.-G. (2025). Hydrogen-free explosive of *N*-azo-bridged 1,2,4-triazole: A novel nitrogen-chain-extended compound with exceptional energy performance. ACS Appl. Mater. Interfaces.

[13] Dong W.-S., Xu M., Tariq Q.-u.-N., Lu Z.-j., Yu Q., Zhang J.-G. (2025). Tailoring azo-bridged nitropyrazoles: Enhancing energy thresholds through complete functionalization. J. Org. Chem..

[14] Xia H., Zhang W., Jin Y., Song S., Wang K., Zhang Q. (2019). Synthesis of thermally stable and insensitive energetic materials by incorporating the tetrazole functionality into a fused-ring 3,6-dinitropyrazolo-[4,3-c]pyrazole framework. ACS Appl. Mater. Interfaces.

[15] Klapoetke T.M., Petermayer C., Piercey D.G., Stierstorfer J. (2012). 1,3-Bis(nitroimido)-1,2,3-triazolate anion, the N-nitroimide moiety, and the strategy of alternating positive and negative charges in the design of energetic materials. J. Am. Chem. Soc..

[16] Dippold A.A., Klapotke T.M. (2013). Synthesis and characterization of 5-(1,2,4-triazol-3-yl)tetrazoles with various energetic functionalities. Chem.-Asian J..

[17] Dalinger I.L., Khoranyan T.E., Suponitsky K.Y., Muravyev N.V., Sheremetev A.B. (2024). Energetic nitrated azole assemblies: Linear alliance of isomeric furazan-1,2,4-triazole-pyrazole combinations. Cryst. Growth Des..

[18] Ma J., Chinnam A.K., Cheng G., Yang H., Zhang J., Shreeve J.M. (2021). 1,3,4-Oxadiazole Bridges: A strategy to improve energetics at the molecular level. Angew. Chem. Int. Ed..

[19] Feng S., Li Y., Lai Q., Cai J., Wang Z., Yin P., He C., Pang S. (2022). A strategy for stabilizing of N8 type energetic materials by introducing 4-Nitro-1,2,3-Triazole scaffolds. Chem. Eng. J..

[20] Liu Y., Zhang X., Li J., Pei X., Pang S., He C. (2025). Construction of bis-heterocyclic energetic compounds via C–N coupling reactions. JACS Au.

[21] Kumar P., Kumar N., Ghule V., Dharavath S. (2024). Zwitterionic fused pyrazolo-triazole based high performing energetic materials. Chem. Commun..

[22] Xu Z., Cheng G., Yang H., Ju X., Yin P., Zhang J., Shreeve J.M. (2017). A facile and versatile synthesis of energetic furazan-functionalized 5-nitroimino-1,2,4-triazoles. Angew. Chem. Int. Ed..

[23] Zhang J., Bi F., Zhai L., Huo H., Yang Z., Wang B. (2020). A comparative study of the structures, thermal stabilities and energetic performances of two energetic regioisomers: 3(4)-(4-aminofurazan-3-yl)-4(3)-(4-nitrofurazan-3-yl)furoxan. RSC Adv..

[24] Wu B., Yang H., Lin Q., Wang Z., Lu C., Cheng G. (2015). New thermally stable energetic materials: Synthesis and characterization of guanylhydrazone substituted furoxan energetic derivatives. New J. Chem..

[25] Yang R., Liu Y., Dong Z., Li H., Ye Z. (2021). 3-R-4-(5-methyleneazide-1,2,4-oxadiazol-3-yl)furazan and its ionic salts as low-sensitivity and high-detonation energetic materials. New J. Chem..

[26] Zheng Y., Qi X., Chen S., Song S., Zhang Y., Wang K., Zhang Q. (2021). Self-assembly of nitrogen-rich heterocyclic compounds with oxidants for the development of high-energy materials. ACS Appl. Mater. Interfaces.

[27] Yu Q., Yang H., Imler G.H., Parrish D.A., Cheng G., Shreeve J.M. (2020). Derivatives of 3,6-bis(3-aminofurazan-4-ylamino)-1,2,4,5-tetrazine: Excellent energetic properties with lower sensitivities. ACS Appl. Mater. Interfaces.

[28] Ma J., Tang J., Yang H., Yi Z., Wu G., Zhu S., Zhang W., Li Y., Cheng G. (2019). Polynitro-functionalized triazolylfurazanate triaminoguanidine: Novel green primary explosive with insensitive nature. ACS Appl. Mater. Interfaces.

[29] Yan T., Ma J., Yang H., Cheng G. (2021). Introduction of energetic bis-1,2,4-triazoles bridges: A strategy towards advanced heat resistant explosives. Chem. Eng. J..

[30] Xu Z., Cheng G., Yang H., Zhang J., Shreeve J.M. (2018). Synthesis and characterization of 4-(1,2,4-triazole-5-yl)furazan derivatives as high-performance insensitive energetic materials. Chem.-Eur. J..

[31] Pandey K., Das P., Khatri M., Kumar D. (2024). N-Methylene-C-linked nitropyrazoles and 1,2,4-triazolone-3-one: Thermally stable energetic materials with reduced sensitivity. Dalton Trans..

[32] Qu Y., Zeng Q., Wang J., Ma Q., Li H., Li H., Yang G. (2016). Furazans with azo linkages: Stable CHNO energetic materials with high densities, highly energetic performance, and low impact and friction sensitivities. Chem.-Eur. J..

[33] Spackman P.R., Turner M.J., McKinnon J.J., Wolff S.K., Grimwood D.J., Jayatilaka D., Spackman M.A. (2021). CrystalExplorer: A program for hirshfeld surface analysis, visualization and quantitative analysis of molecular crystals. J. Appl. Crystallogr..

[34] Johnson E.R., Keinan S., Mori-Sánchez P., Contreras-García J., Cohen A.J., Yang W. (2010). Revealing noncovalent interactions. J. Am. Chem. Soc..

[35] Lu T. (2021). Simple, reliable, and universal metrics of molecular planarity. J. Mol. Model..

[36] Lu T., Chen F. (2012). Quantitative analysis of molecular surface based on improved marching tetrahedra algorithm. J. Mol. Graph. Model..

[37] Lu T., Chen Q. (2020). A simple method of identifying π orbitals for non-planar systems and a protocol of studying π electronic structure. Theor. Chem. Acc..

[38] Wang X., Liu Z., Wang J., Lu T., Xiong W., Yan X., Zhao M., Orozco-Ic M. (2023). Electronic structure and aromaticity of an unusual cyclo[18]carbon precursor, C18Br6. Chem.-Eur. J..

