# Supplementary material for: Synthesis, Characterization, and Properties of Novel Coplanar Bicyclic Compounds Based on Triazolofurazane Compounds
Source: Molecules. 2025 Jun 29;30(13):2803. doi: 10.3390/molecules30132803 (PMC12250748; doi:10.3390/molecules30132803)
Supplement: Supplementary file 1 [file molecules-30-02803-s001.zip › molecules-3713956-supplementary.pdf]

Supporting Information

Synthesis, Characterization, and Properties of Novel Coplanar Bicyclic Compounds Based on Triazolofurazane Compounds

Mei-Qi Xu, Wen-Shuai Dong, Qamar-un-Nisa Tariq, Chao Zhang, Cong Li, Zu-Jia Lu, Bin-Shan Zhao, Qi-Yao Yu * and Jian-Guo Zhang *

State Key Laboratory of Explosion Science and Technology, Beijing Institute of Technology, Beijing 100081, China; xumeiqi1674@163.com (M.-Q.X.); dws08292020@163.com (W.-S.D.); qamarnisha@yahoo.com (Q.-u.-N.T.); 18810109953@126.com (C.Z.); l18656815056@163.com (C.L.); 15201657211@163.com (Z.-J.L.); 18810591681@163.com (B.-S.Z.)

* Correspondence: qiyaoyu@bit.edu.cn (Q.-Y.Y.); zjgbit@bit.edu.cn (J.-G.Z.); Tel.: +86-10-68918091 (J.-G.Z.)

Table of Contents

Section S1. Crystal structure data 1

Section S2. Theoretical calculation method of formation enthalpy 3

Section S3. Intermolecular interactions 4

Section S4. ^{1}H and ^{13}C NMR spectra for all compounds 6

Section S5. IR spectra of all compounds 9

Section S6. Noncovalent interaction analysis 11

Section S1. Crystal structure data

Table S1 Bond Lengths for **2**·Ca^{2+}·$6H_2O$.

Atom	Length/Å	Atom	Length/Å
Ca1-O4	2.643(4)	N7-N6	1.306(5)
Ca1- O5	2.316(3)	N7-O3	1.240(5)
Ca1-O7	2.429(4)	N6-C1	1.363(6)
Ca1-N7	3.013(4)	N2-N3	1.359(5)
Ca1-N6	2.477(4)	N2-C2	1.353(6)
Ca1-O8	2.296(3)	N4-C015	1.317(6)

Ca1-N1	2.545(4)	N1-C017	1.392(6)
Ca1-O6	2.295(4)	N1-C2	1.364(6)
O4-N7	1.282(5)	C015-C017	1.454(7)
O2-C2	1.283(6)	C015-C1	1.438(6)
O1-N4	1.373(5)	N5-C1	1.308(6)
O1-N5	1.401(5)	C017-N3	1.315(6)

Table S2 Bond Angles for **2**$\cdot Ca^{2+} \cdot 6H_2O$.

Atom	Angle/°	Atom	Angle/°
O4-Ca1-N7	25.13(10)	N4-O1-N5	110.8(3)
O5-Ca1-O4	86.62(12)	O4-N7-Ca1	61.1(2)
O5-Ca1-O7	101.42(13)	O4-N7-N6	114.4(4)
O5-Ca1-N7	81.33(13)	N6-N7-Ca1	53.8(2)
O5-Ca1-N6	81.29(13)	O3-N7-Ca1	172.3(3)
O5-Ca1-N1	86.82(12)	O3-N7-O4	121.0(4)
O7-Ca1-O4	152.77(12)	O3-N7-N6	124.6(4)
O7-Ca1-N7	176.85(11)	N7-N6-Ca1	101.0(3)
O7-Ca1-N6	156.28(14)	N7-N6-C1	118.7(4)
O7-Ca1-N1	83.65(12)	C1-N6-Ca1	138.6(3)
N6-Ca1-O4	50.17(12)	C2-N2-N3	111.2(4)
N6-Ca1-N7	25.19(12)	C015-N4-O1	106.3(4)
N6-Ca1-N1	72.92(13)	C017-N1-Ca1	132.1(3)
O8-Ca1-O4	86.88(12)	C2-N1-Ca1	125.0(3)
O8-Ca1-O5	169.49(13)	C2-N1-C017	101.9(4)
O8-Ca1-O7	88.09(13)	N4-C015-C017	120.2(4)
O8-Ca1-N7	89.29(12)	N4-C015-C1	108.3(4)
O8-Ca1-N6	88.21(13)	C1-C015-C017	131.4(4)
O8-Ca1-N1	89.82(13)	C1-N5-O1	105.3(4)
N1-Ca1-O4	123.05(12)	N1-C017-C015	123.2(4)
N1-Ca1-N7	98.08(12)	N3-C017-N1	115.2(4)
O6-Ca1-O4	76.99(12)	N3-C017-C015	121.7(4)
O6-Ca1-O5	88.36(13)	C017-N3-N2	102.9(4)
O6-Ca1-O7	77.29(12)	N6-C1-C015	119.9(4)
O6-Ca1-N7	101.38(13)	N5-C1-N6	130.8(5)
O6-Ca1-N6	126.43(13)	N5-C1-C015	109.3(4)
O6-Ca1-O8	98.21(13)	O2-C2-N2	124.3(5)
O6-Ca1-N1	158.99(14)	O2-C2-N1	126.8(5)
N7-O4-Ca1	93.8(3)	N2-C2-N1	108.9(4)

Table S3 Bond Lengths for **3**·MeOH.

Atom	Length/Å	Atom	Length/Å
O1-N5	1.404(4)	N5-C1	1.320(5)
O1-N4	1.387(4)	C4-N1	1.372(5)
O2-C4	1.252(5)	N1-C3	1.360(5)
O4-N7	1.249(4)	N3-C3	1.308(5)
O3-N7	1.274(5)	C3-C2	1.455(6)
N7-N6	1.328(4)	N4-C2	1.304(5)
O5-C5	1.411(5)	N6-C1	1.369(5)
N2-C4	1.351(5)	C2-C1	1.446(5)
N2-N3	1.379(4)		

Table S4 Bond Angles for **3**·MeOH

Atom	Angle/°	Atom	Angle/°
N4-O1-N5	111.2(3)	N1-C3-C2	123.5(4)
O4-N7-O3	121.3(4)	N3-C3-N1	112.3(4)
O4-N7-N6	123.9(4)	N3-C3-C2	124.2(4)
O3-N7-N6	114.8(3)	C2-N4-O1	105.4(3)
C4-N2-N3	113.0(3)	N7-N6-C1	116.7(3)
C1-N5-O1	105.2(3)	N4-C2-C3	120.8(4)
O2-C4-N2	128.3(4)	N4-C2-C1	109.9(4)
O2-C4-N1	127.9(4)	C1-C2-C3	129.3(4)
N2-C4-N1	103.7(3)	N5-C1-N6	132.3(4)
C3-N1-C4	107.7(3)	N5-C1-C2	108.2(4)
C3-N3-N2	103.2(3)	N6-C1-C2	119.5(4)

Section S2. Theoretical calculation method of formation enthalpy

Both **2** and **3** were subjected to geometric optimization and vibration analysis at the B3PW91/6-31G** level, and higher precision single point energy calculations were performed again at M062X/def2TZVP. Add the high-precision single point energy and the enthalpy correction obtained from vibration analysis to obtain the corresponding enthalpy of the structure. The calculation principle of the enthalpy of molecular formation is as follows:

$$aC + \frac{b}{2}H_2 + \frac{c}{2}N_2 + \frac{d}{2}O_2 \rightarrow C_aH_bN_cO_d$$

By definition, the enthalpy of formation is the reaction enthalpy of the above equation. The enthalpy of reaction is determined by calculating the difference between the enthalpy of the products and the enthalpy of the reactants. To calculate the enthalpy of each species involved in the reaction,

quantum chemistry computational programs such as Gaussian or ORCA can be used. Carbon is a solid at room temperature, but the enthalpy value obtained from quantum chemistry calculation corresponds to its formation in the gaseous state. Subtracting the sublimation enthalpy ($H_{sub}(C)$) from the enthalpy value ($H(C)$) obtained from quantum chemistry calculation is necessary to determine the enthalpy of carbon in its stable phase (solid). In summary, the formula for calculating the enthalpy of formation using the definition method is as follows:

$$\Delta_f H_m^{\theta} = \Delta_r H_m^{\theta} = H(C_aH_bN_cO_d) - a[H(C) - H_{sub}(C)] - \frac{b}{2}H(H_2) - \frac{c}{2}H(N_2) - \frac{d}{2}H(O_2)$$

All the enthalpy values provided above are enthalpies at 298 K. The enthalpy represented by $H(C_aH_bN_cO_d)$corresponds to the enthalpy of a single molecule. Therefore, the final result denotes the gas-phase enthalpy of formation of the compound at 298 K.

For the formation reaction, the gas-phase heat of reaction at 298 K can be calculated from the following equation:

$$\Delta_f H(C_aH_bN_cO_d) = a\Delta_f H(C) + b\Delta_f H(H) + c\Delta_f H(N) + d\Delta_f H(O) - \Delta H_{atomization}$$

Where $\Delta_f H(C)$, $\Delta_f H(H)$, $\Delta_f H(N)$ and $\Delta_f H(O)$ are experimental gas phase enthalpies of formation of C, H, N and O atoms; $\Delta H_{atomization}$ is standard reaction enthalpy for atomization reaction, which can be can be calculated by the following equation:

$$\Delta H_{atomization} = aH(C) + bH(H) + cH(N) + dH(O) - H(C_aH_bN_cO_d)$$

According to Hess law, the solid-phase enthalpy of formation can be obtained from the gas-phase enthalpy of formation of and the enthalpy of sublimation as follows:

$$\Delta H_{solid} = \Delta H_{gas} - \Delta H_{sublimation}$$

In this work, we obtained the enthalpy of sublimation using the method of ref. S1:

$$\Delta H_{sublimation} = a(SA)^2 + b\sqrt{\upsilon\sigma_{tot}^2} + c$$

Where SA is molecular surface area, υ is equilibrium constant of positive and negative electrostatic potentials at the surface of the molecule and σ_{tot} is variance of total surface electrostatic potential.

Section S3. Intermolecular interactions

To further analyze the distribution of hydrogen bonds and π-π interactions in the compounds, the Hirshfeld surface and 2D fingerprint plots were generated and examined using CrystalExplorer software, highlighting the percentage contribution of each atom (see Figure 3). The Hirshfeld surface is a powerful tool for identifying intermolecular interactions within crystal structures and provides

effective graphical visualization.

Based on the electron cloud density, the interactions between the selected molecule and its surrounding molecules are represented on a contact surface. Areas shaded in red indicate regions of high electron density, suggesting the presence of hydrogen bonds. Conversely, blue areas signify low electron density, indicating the absence of significant intermolecular interactions. The Hirshfeld surface represents a three-dimensional pattern, while the 2D fingerprint serves as a two-dimensional representation of the Hirshfeld surface. Generally, the peak corresponding to intermolecular O/N···H interactions is observed in the lower-left section of the fingerprint (hydrogen bond donor), while the peak associated with H···O/N interactions is found in the lower right section (hydrogen bond acceptor). In the compounds **2**·Ca^{2+}·$6H_2O$ and **3**·MeOH, the coplanar cation conjugation effect has led to the observation of numerous red plaques on the edges of the cation plate-like Hirshfeld surface in both crystals, indicating the presence of strong hydrogen bonds within these crystal structures. As illustrated in Figures 3a to 3c, the highest proportion of O···H hydrogen bonds in the crystal structure of **2**·Ca^{2+}·$6H_2O$ is 39.9%, while the highest proportion of N···H hydrogen bonds is 17.2%, resulting in a total hydrogen bond proportion of 57.1%. In the case of the crystal structure of **3**·MeOH, as shown in Figures 3d to 3f, the highest proportion of O···H hydrogen bonds is 44.1%, the highest proportion of N···H hydrogen bonds is 21.7%, resulting in a combined total of 65.8% for hydrogen bonds. For multi-nitrogen heterocyclic energetic compounds, interactions such as C···O, C···N, N···N, O···O, and O···N typically signify π-π and p-π stacking interactions. Notably, in the crystal structure of **3**·MeOH, the proportion of O···N interactions as a contribution to the main weak interactions is 8.9%, while C···N interactions account for 5.7%. This suggests that there is a significant presence of π-π and p-π interactions within the crystal assembly of **3**·MeOH.

Section S4. ^{1}H and ^{13}C NMR spectra for all compounds

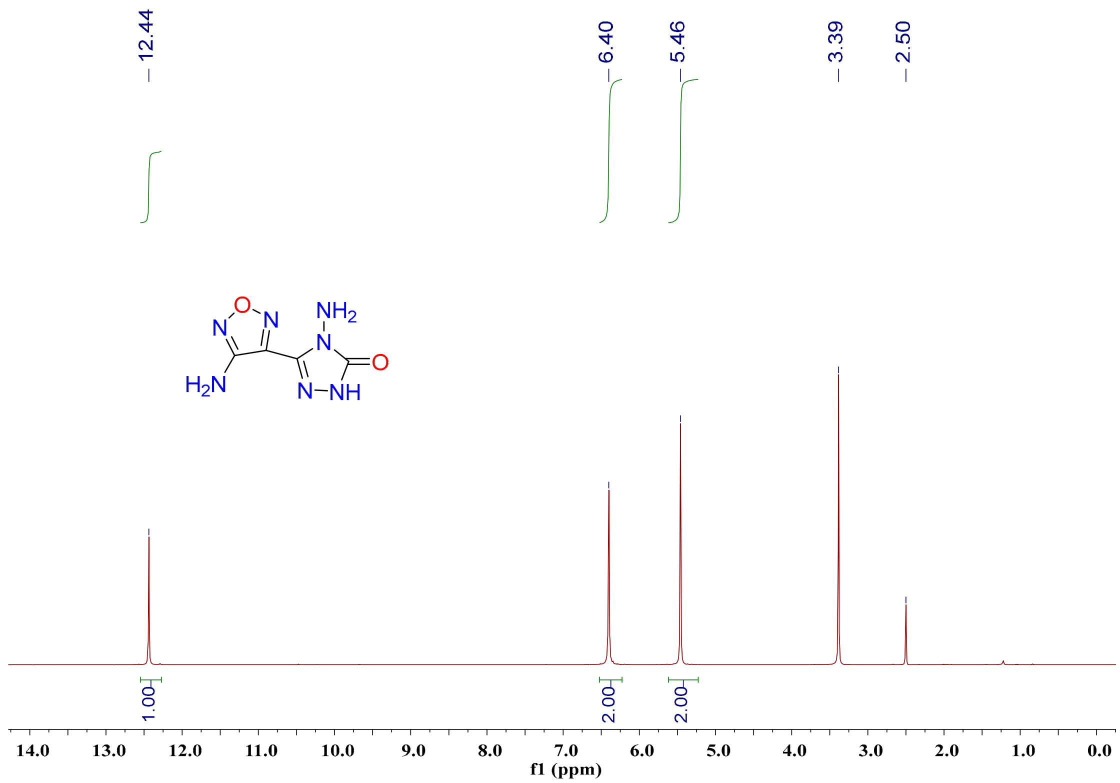

Figure S1. ^{1}H NMR spectrum of 4-amino-5-(4-amino-1,2,5-oxadiazol-3-yl)-2,4-dihydro-3*H*-1,2,4-triazol-3-one (**1**) in DMSO-d_6 at 400 MHz.

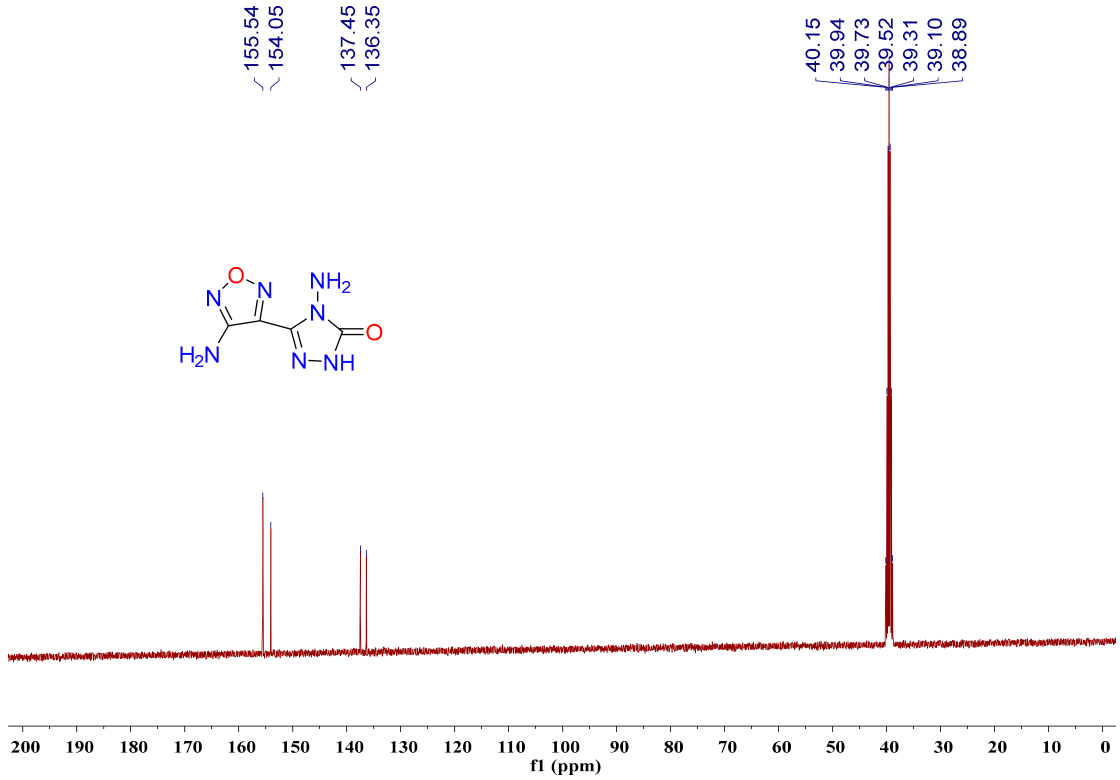

Figure S2. ^{13}C NMR spectrum of 4-amino-5-(4-amino-1,2,5-oxadiazol-3-yl)-2,4-dihydro-3*H*-

1,2,4-triazol-3-one (**1**) in DMSO-d_6 at 101 MHz.

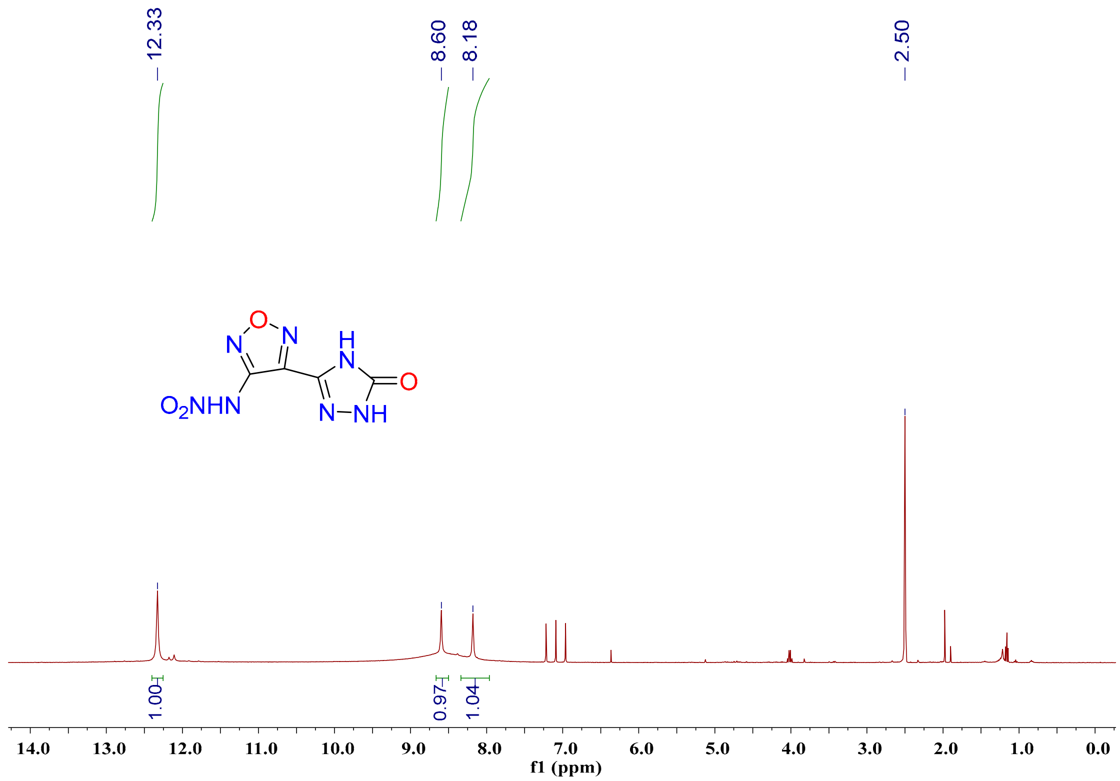

Figure S3. ^{1}H NMR spectrum of *N*-(4-(5-oxo-4,5-dihydro-1*H*-1,2,4-triazol-3-yl)-1,2,5-oxadiazol-3-yl)nitramide (**2**) in DMSO-d_6 at 400 MHz.

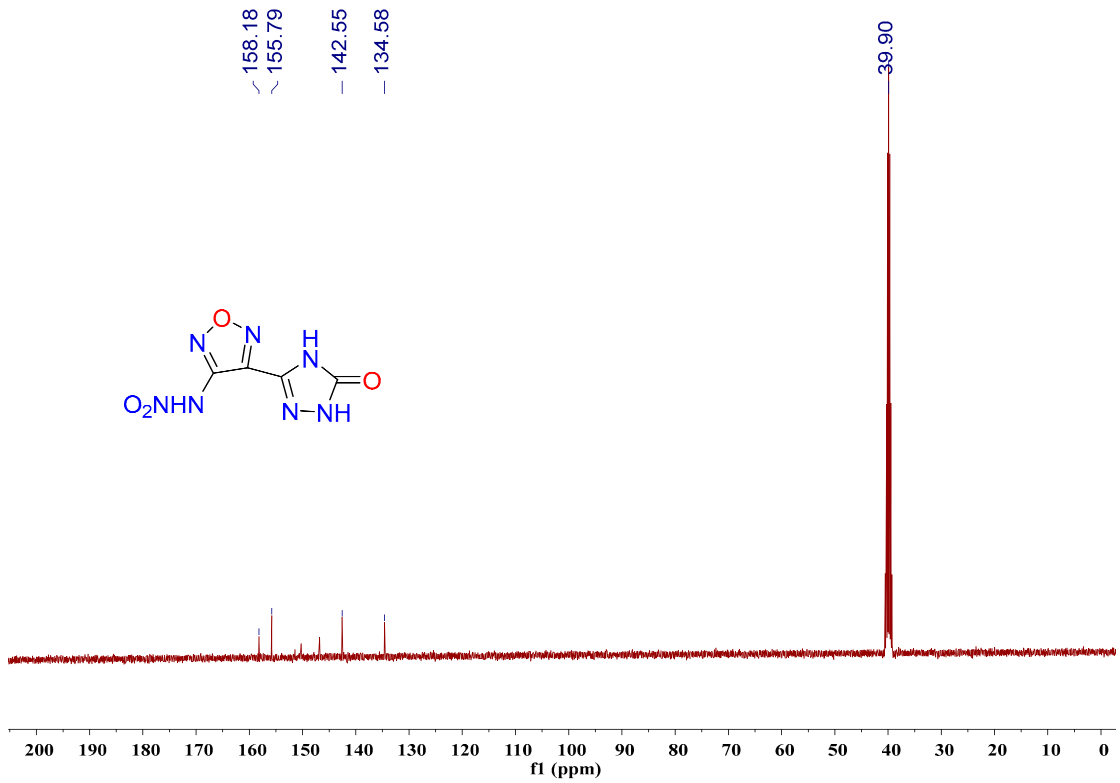

Figure S4. ^{13}C NMR spectrum of *N*-(4-(5-oxo-4,5-dihydro-1*H*-1,2,4-triazol-3-yl)-1,2,5-

oxadiazol-3-yl)nitramide (**2**) in DMSO-d_6 at 101 MHz.

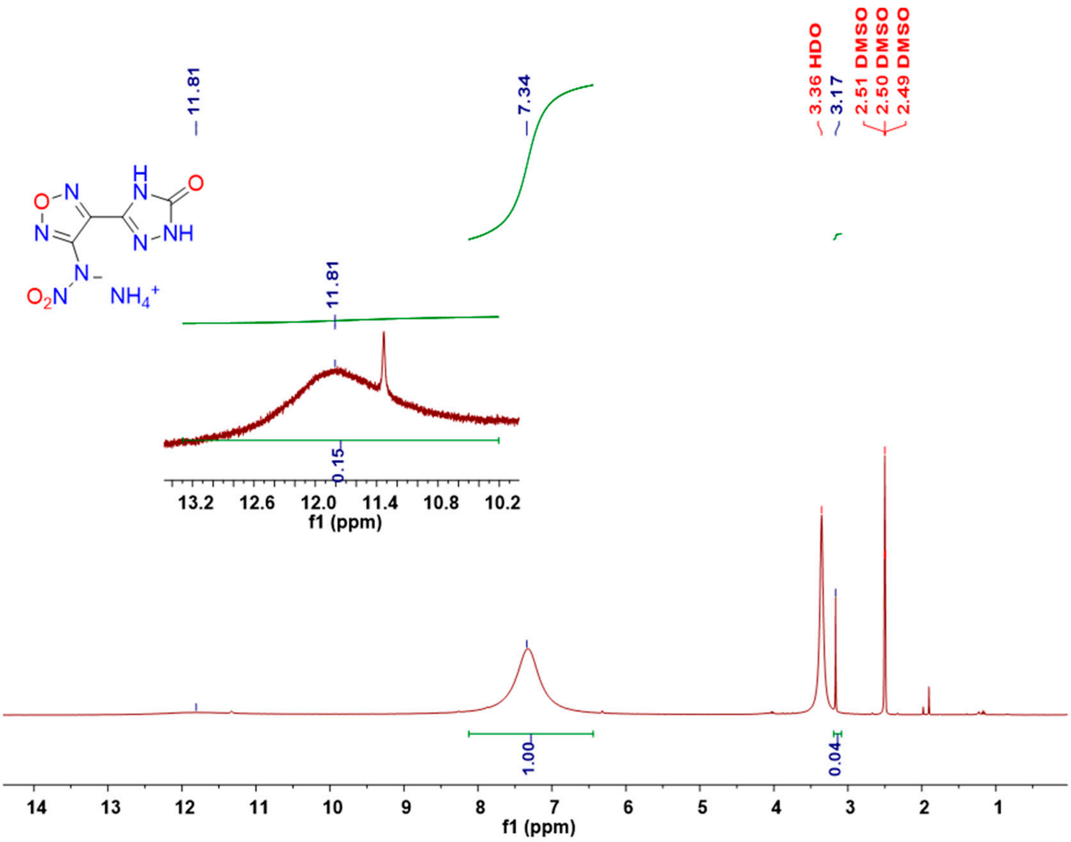

Figure S5. ^{1}H NMR spectrum of ammonium nitro(4-(5-oxo-4,5-dihydro-1H-1,2,4-triazol-3-yl)-1,2,5-oxadiazol-3-yl)amide (**3**) in DMSO-d_6 at 400 MHz.

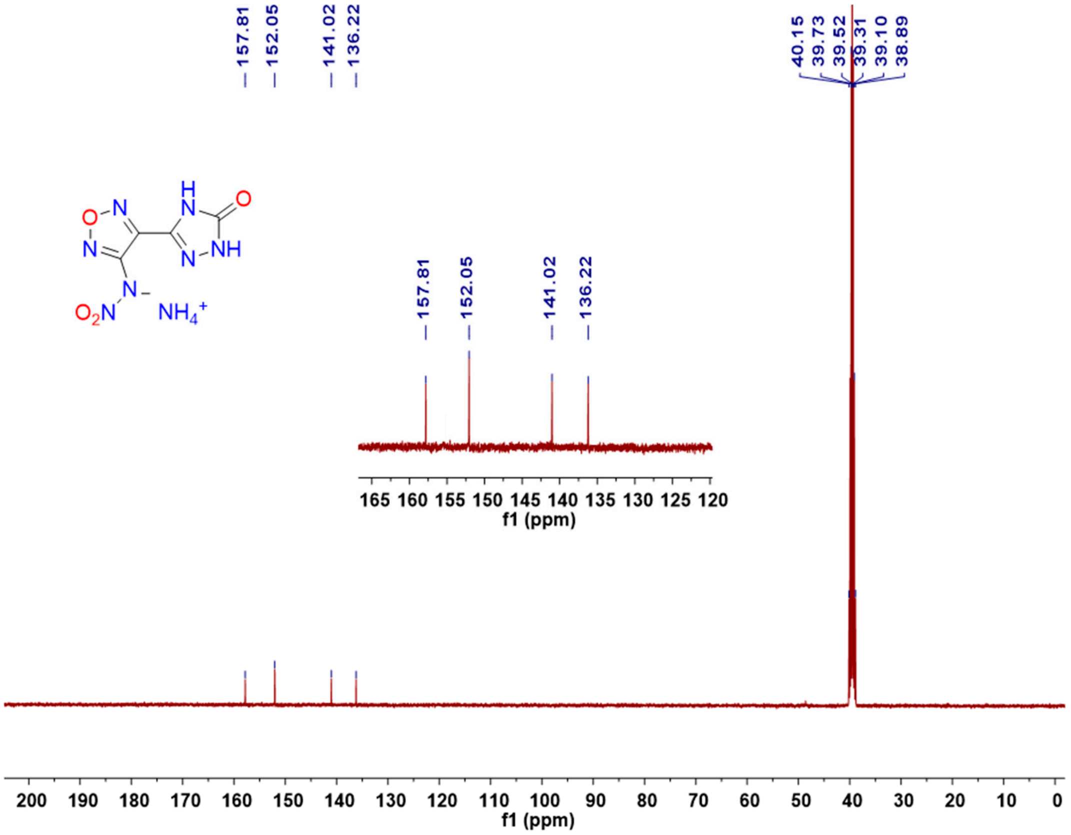

Figure S6. ^{13}C NMR spectrum of ammonium nitro(4-(5-oxo-4,5-dihydro-1H-1,2,4-triazol-3-yl)-1,2,5-oxadiazol-3-yl)amide (**3**) in DMSO-d_6 at 101 MHz.

Section S5. IR spectra of all compounds

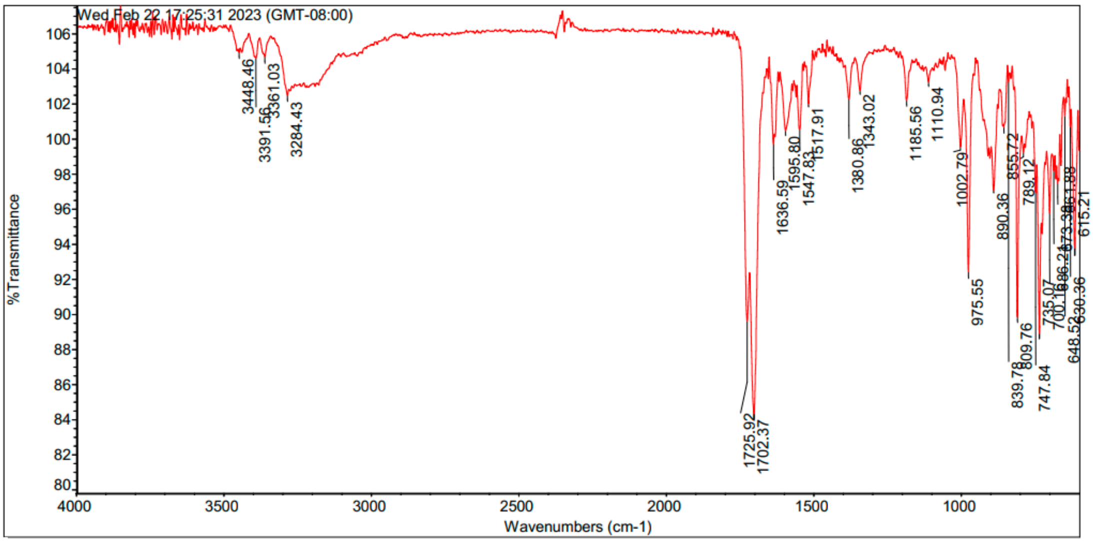

Figure S7. IR spectrum for 4-amino-5-(4-amino-1,2,5-oxadiazol-3-yl)-2,4-dihydro-3*H*-1,2,4-triazol-3-one (**1**)

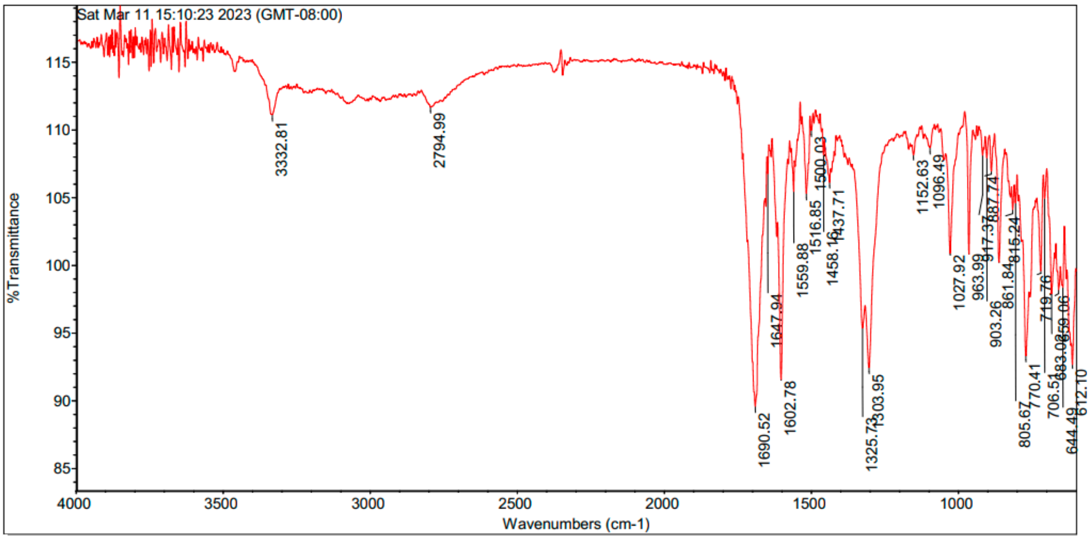

Figure S8. IR spectrum for ***N*-(4-(5-oxo-4,5-dihydro-1*H*-1,2,4-triazol-3-yl)-1,2,5-oxadiazol-3-yl)nitramide** (**2**)

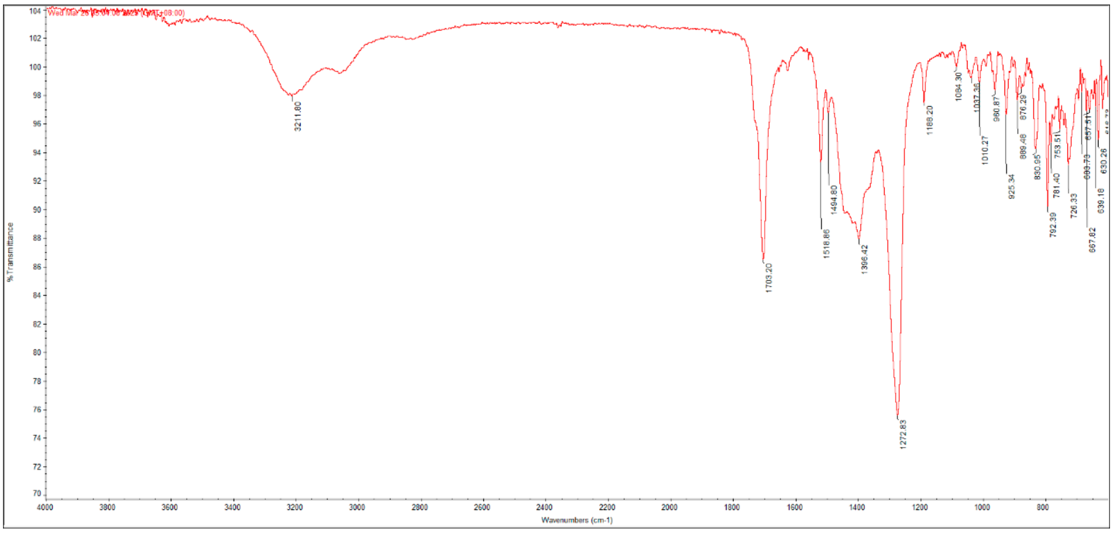

Figure S9. IR spectrum for ammonium nitro(4-(5-oxo-4,5-dihydro-1H-1,2,4-triazol-3-yl)-1,2,5-oxadiazol-3-yl)amide (**3**)

Section S6. Noncovalent interaction analysis

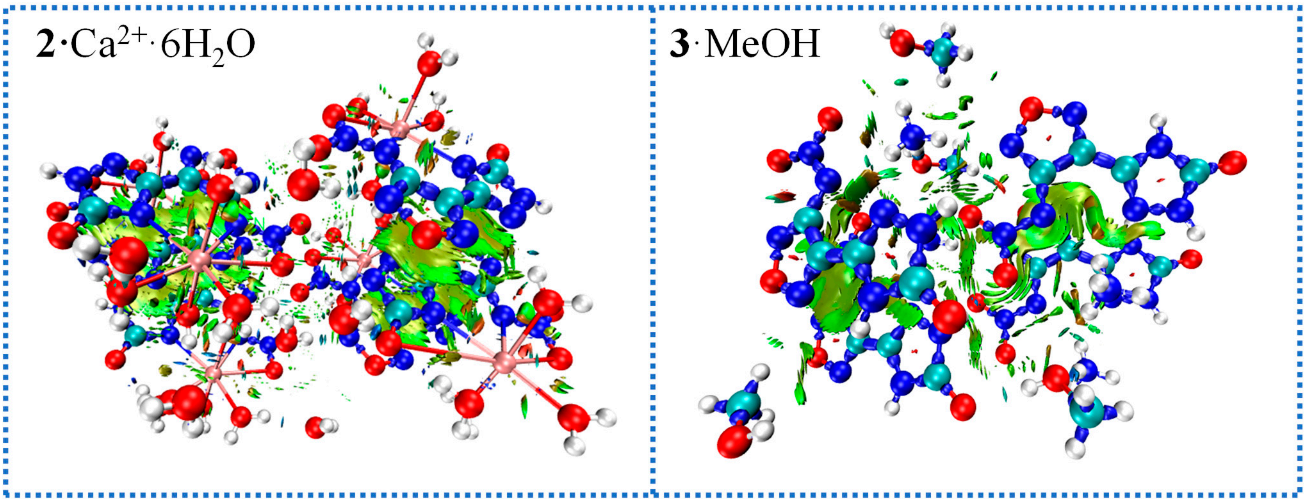

Figure S10. Noncovalent interaction analysis for **2**·Ca^{2+}·6H_2O (left) and **3**·MeOH (right)
